# SparcleQC: Automated Input File Creation for QM/MM Studies of Protein:Ligand Complexes

**DOI:** 10.1021/acs.jcim.5c00617

**Published:** 2025-06-17

**Authors:** Caroline S. Glick, Isabel P. Berry, C. David Sherrill

**Affiliations:** Center for Computational Molecular Science and Technology, School of Chemistry and Biochemistry, and School of Computational Science and Engineering, 1372 Georgia Institute of Technology, Atlanta, Georgia 30332-0400 United States

## Abstract

SparcleQC is a Python package that, given a protein:ligand complex in the Protein Data Bank (PDB) file format, can create quantum mechanics/molecular mechanics (QM/MM)-like input files for the electronic structure theory packages Psi4, Q-Chem, and NWChem. The resulting input files include quantum mechanical representations of the ligand and a small section of the protein, surrounded by point charges that represent the rest of the protein. Creation of these QM/MM input files includes cutting and capping the QM subregion, obtaining point charges for the protein, and adjusting charges at the QM/MM boundary; and each of these tasks are automated by the software. In this article, we describe the details of SparcleQC’s procedure, show examples of the Python API, and explain additional features that are helpful in protein:ligand interaction studies. Finally, we show that SparcleQC enables automated preparation of input files for QM/MM calculations, which can return can return accurate interaction energies in minutes, while a fully quantum mechanical computation on the protein:ligand complex could take days, if it is even possible.

## Graphical abstract

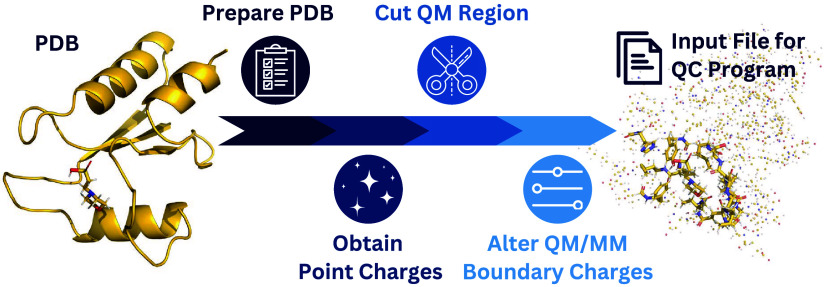

## Introduction

The steep computational cost of quantum mechanics (QM) methods often prohibits their use for systems with more than a few hundred atoms. Mixed quantum mechanics/molecular mechanics (QM/MM) methods enable the study of larger systems by dividing them into a QM subsystem of a reasonable size according to the ab initio method chosen, and an MM subsystem.^1,2^ The QM/MM energy of a system is then

1 $$E=E_{\mathrm{QM}}+E_{\mathrm{MM}}+E_{\mathrm{QM}/\mathrm{MM}}$$

where *E*_QM_ is the QM energy of the QM subsystem, *E*_MM_ is the MM energy of the MM subsystem, and *E*_QM/MM_ is the interaction energy between the two systems. Popular approximations of *E*_QM/MM_ include electrostatic^3^ and polarizable^2^ embedding. Electrostatic embedding accounts for the polarization of the QM region by the MM subsystem, whereas in polarizable embedding, the MM region is also polarized by the QM region.

QM/MM has been widely used to study dynamic processes and static properties, like proton transfer reactions^4–6^ and protein:ligand interaction energies.^7–9^ Its popularity has led to the support of QM/MM within many electronic structure theory packages, providing direct access to many ab initio methods for modeling the QM region. For example, our group has recently expanded symmetry-adapted perturbation theory (SAPT),^10,11^ a method that directly computes interaction energies (the attraction or repulsion between two molecules), to include the effect of external point charges in Psi4.^12^ In addition to returning the interaction energy, SAPT returns the four physically relevant interaction components of electrostatics, exchange-repulsion, induction/polarization, and London dispersion for systems like solvated dimers and protein: ligand complexes.

Many types of chemical systems can be studied with QM/MM, but systems where the QM and MM subsystems are from the same molecule pose special challenges to the setup of these calculations. In this paper, we present software to automate input file preparation specifically for protein: ligand systems where the separation of QM and MM regions involves cutting through covalent bonds in the protein. Proteins typically contain thousands of atoms and, due to their three-dimensional and folded nature, tens of bonds may need to be cut to separate the protein into QM and MM regions, making for an especially tedious process.

When representing a protein with both QM and MM, special precautions must be taken to control the errors induced by “cutting” the protein. Normally, carbon–carbon bonds are severed due to their nonpolar and single-bond characteristics.^13,14^ This is shown in the top panel of Figure 1. The QM region must then be capped, often with a hydrogen link atom.^13,14^ Once the QM region is defined and capped, all other atoms are represented by point charges and become the MM subsystem. The point charges may come from classical force fields, such as CHARMM^15^ or Amber.^16,17^

**Figure 1:**
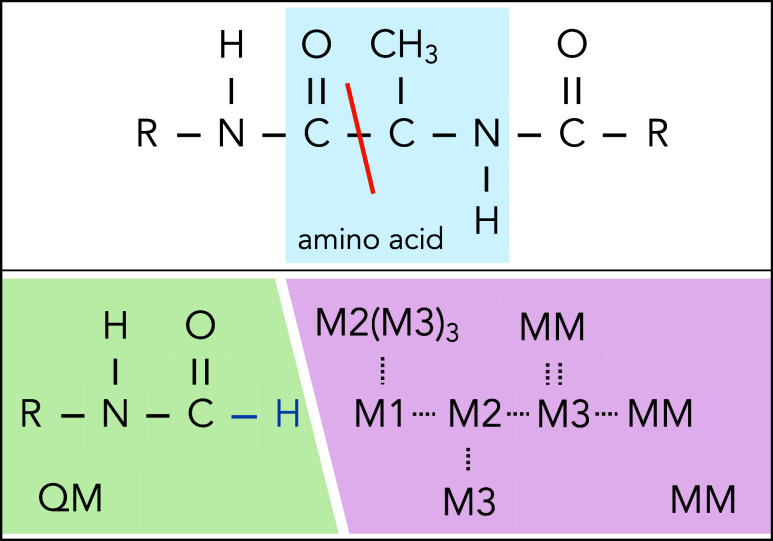
(Top) Representative chain of amino acids with one amino acid highlighted in blue. The orange line shows the carbon–carbon bond that would be cut to separate this system into QM and MM subsystems. (Bottom) Protein divided into QM and MM subsystems. The QM subsystem is capped with a hydrogen atom (blue). The atoms in the MM region are now represented by point charges, and they are named according to distance from the cut bond.

Placing all point charges at the original atomic coordinates can cause overpolarization of the QM region due to the closeness of the MM point charges to the capped QM subsystem. To reduce this overpolarization, many charge schemes exist, which alter the locations of the charges and the charge values themselves (another tedious task). Of the existing charge schemes, there is no general consensus on which is best, lending to further research of benchmarking these schemes on various systems and calculation-types. For example, there are nine charge schemes that we have previously tested in ref 7., and these are all supported by SparcleQC and explained in the Methods section.

The process of preparing QM/MM input files has benefited from existing software.^18–23^ However, of these packages, few can handle a QM/MM region that crosses a covalent boundary. Of those that can handle these covalent junctions, many require working with a graphical user interface (GUI), which prohibits any high throughput and fully automatic workflow. Furthermore, to our knowledge, no existing packages allow for the creation of Psi4 or Q-Chem^24^ input files.

Here, we present SparcleQC, a Python package for automatically creating QM/MM-like input files of protein:ligand complexes for Psi4, Q-Chem, and NWChem.^25^ The minimum input required for SparcleQC is a protein:ligand PDB file and a Python dictionary object of user-defined options, like the desired force field, electronic structure theory software, size of the QM system, etc. SparcleQC can then generate a QM/MM protein:ligand input file without any additional user interaction. SparcleQC is open-source and freely available through GitHub.

## Methodology

### Basic Input and Capabilities

SparcleQC follows the basic flow presented in Figure 2. The required input includes a Python dictionary, which can be given as a SparcleQC input file or as a Python object, and a PDB file of the protein: ligand complex. SparcleQC automatically adds *N*-methylamide (NME) and acetyl group (ACE) end-caps with PyMOL’s API^26^ unless the PDB is precapped. The program will then obtain point charges for the nonligand atoms (protein, solvent, and ions) using AmberTools. If CHARMM force fields are more desirable, the user may upload CHARMM-GUI^23^ topology files, and the charges from the those files will be used. Users can specify water force fields, or provide their own charges for three- and four-point water models.

**Figure 2:**
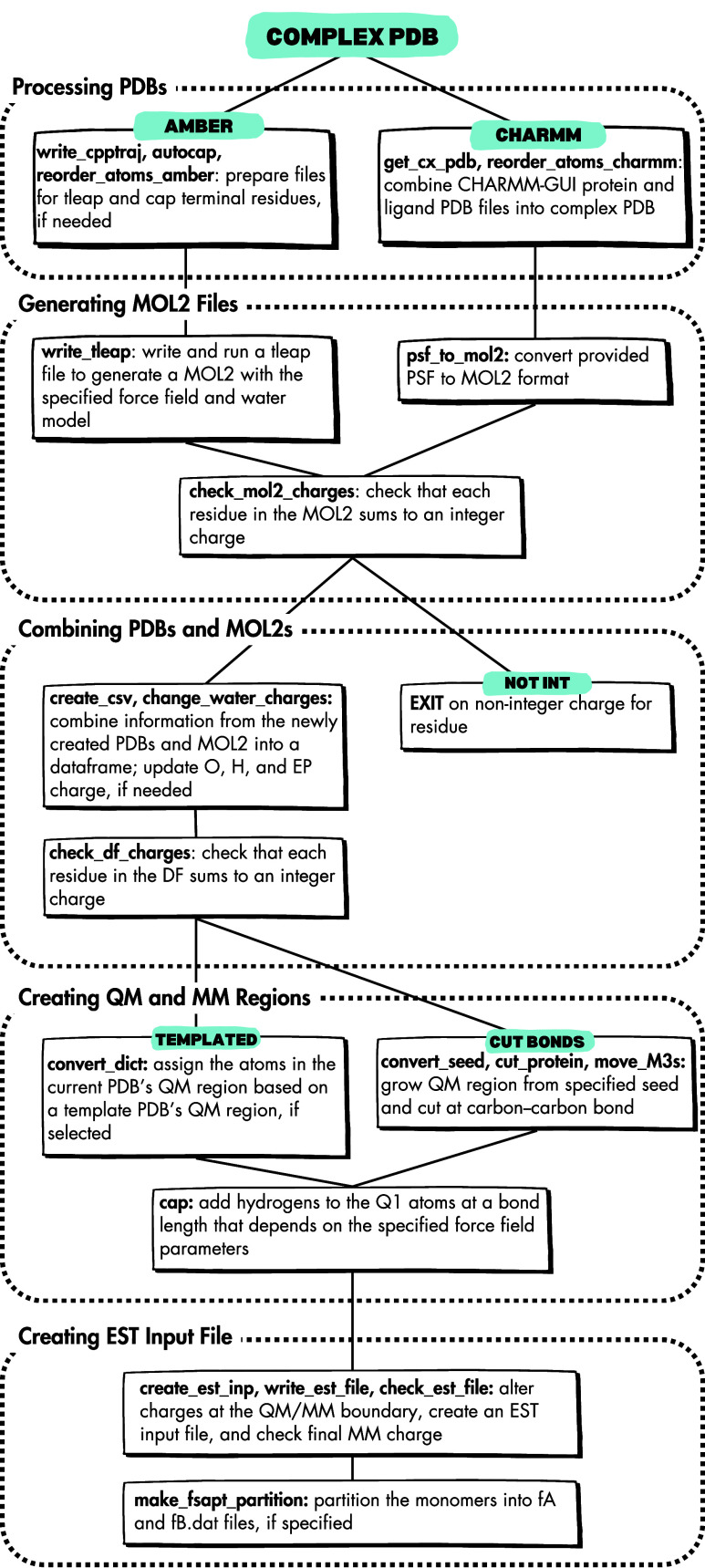
General procedure carried out by SparcleQC. Within each solid white box, function names are in bold and are followed by descriptions.

SparcleQC writes all nonligand point charges and coordinates to a MOL2 file, then checks for integer charges of residues (a property of CHARMM and Amber force fields). Residues with noninteger charges indicate PDB errors (e.g., missing atoms or improper naming of residues), which the program reports for correction. Correcting the PDB file and restarting SparcleQC will remedy the issue. Relevant data is then stored in a Pandas dataframe.

SparcleQC uses PyMOL’s API to define the QM region by selecting whole residues that have an atom within the user-defined distance (given in Angstroms) of the user-defined ligand atom. We refer to this ligand atom as a “seed”, in analogy to a nucleation site from which the QM region “grows”. Users may also declare their seed to be the whole ligand. The region expands or shrinks to terminate at the nearest carbonyl carbon -α carbon bond (like in Figure 1). To prevent steric clashes between capping hydrogens, SparcleQC detects cases where multiple QM/MM cuts occur in close sequence proximity. In such cases, it iteratively expands the QM region to absorb overlapping boundary residues. Once the composition of the QM subsystem is finalized, a dictionary is made that assigns each unique PDB atom ID number to one of six categories: QM, Q1_*bond*, M1_*bond*, M2_*bond*, M3_*bond*, or MM. M1, M2, and M3 correspond to atom labels in Figure 1. Q1 refers to the carbon that is capped with a hydrogen. Special atoms assignments of Q1, M1, M2, and M3 are given for each cut bond, *bond*. The QM and MM categories include all atoms in the QM or MM regions not already assigned to Q1, M1, M2, or M3. All nonligand atoms in the PDB are accounted for in this dictionary, which helps keep track of how close atoms are to each cut bond. This is important for automating the manipulation of boundary charges.

Furthermore, the final QM subsystem must be capped. While there exist packages to automatically add hydrogens,^27^ we would like to ensure that the hydrogens are placed along the cut bond to reduce the additional degrees of freedom introduced to the system. We choose to implement the hydrogen-capping method of Truhlar and co-workers, where the hydrogen is placed along the Q1–M1 bond and the Q1–H bond distance, *r*_Q1–H1_, depends on force field bond distance parameters^28^

2 $$r_{\mathrm{Q1-H1}}=r_{\mathrm{Q1-M1}}\frac{r_{\mathrm{Q1-HL}}^{\mathrm{MM}0}}{r_{\mathrm{Q1-M1}}^{\mathrm{MM}0}}$$

where *r*_Q1–M1_ is the Q1–M1 bond distance, and *r*_Q1–HL_^MM0^ and *r*_Q1–M1_^MM0^ are the bond distance parameters for Q1–HL and Q1–M1, respectively, of the chosen force field.

At this point, if one were to create an electronic structure theory input file of the capped QM region and the point charges of the remaining atoms, the calculation would either (a) present an error due to clashing of the hydrogen link atoms and the M1 charges, or (b) return an energy contaminated by the overpolarization of the QM region by the closest point charges. Therefore, the user has nine options, so-called charge schemes, to handle charges at the boundary of the QM/MM region. SparcleQC creates a quantum chemistry input file that includes the QM and the MM regions with charges altered according to the charge scheme chosen by the user. These are shown in Table S-1 using notation of Figure 1.

The first three charge schemes, Z1,^29^ Z2,^30^ and Z3,^30^ are classified as elimination charge schemes. These simply zero out charges at the boundaries of the QM/MM region, which can lead to a noninteger charge of the MM region. To maintain the MM region’s integer charge, a “distributed” or “balanced” scheme can be used, like the last six schemes in Table S-1.^5,31^ Here, *q*_bal_, the charge needed to return each cut residue to its original integer charge, is distributed. Exactly where this *q*_bal_ is added is what differentiates these six schemes from each other. After charges are altered according to the user-selected scheme, the total charge of the MM region is then printed for confirmation. If a redistributed or balanced scheme is chosen, the MM region should have an integer charge. Additionally, the number of QM atoms in the file is returned for an estimate of computational intensity.

Generally, three input files for the requested electronic structure theory package will be produced. The three files include: (1) the ligand only (QM), (2) the protein only (QM/MM), and (3) the protein:ligand complex (QM/MM). This prepares users for a supermolecular interaction energy calculation, where the separate protein (*E*_P_) and ligand (*E*_L_) energies are subtracted from the complex (*E*_P:L_) energy

3 $$E_{\mathrm{int}}=E_{P:L}-E_{P}-E_{L}$$

For this use case, we also include the option to include the appropriate ghost atoms for the counterpoise correction of Boys and Bernardi.^32^ If symmetry-adapted perturbation theory (SAPT) is listed as the QM method, only one input file will be created. SAPT *directly* computes interaction energies, meaning that an interaction energy is returned in one calculation, rather than three in the supermolecular case.

### Interoperability

SparcleQC creates input files for Psi4, Q-Chem, and NWChem. Default options are set such that a user can create a working input file for these three quantum chemistry programs without setting many explicit options. For users who wish to be more involved, additional options specific to the QC program may be set and/or overwritten for each program in the SparcleQC input file. The original intent was for SparcleQC to work with the Molecular Sciences Software Institute’s (MolSSI)^33^ QCArchive^34^ ecosystem. Within QCArchive, QCEngine^35^ can execute many quantum chemistry codes using a single QCSchema formatted input file. At the time of this writing, QCSchema does not support external charges, but we look forward to working with QCArchive as soon as this feature is implemented, allowing for even more interoperability among quantum chemistry programs.

### Additional Features

In addition to creating QM/MM input files, SparcleQC offers features for studying protein:ligand interactions. Within Psi4, functional group SAPT (F-SAPT)^36^ exists to break down the interaction energy, not only in terms of the four physically relevant components, but also in terms of contributions from the different functional groups within the system. Obtaining a functional-group breakdown of SAPT energies requires that the user specify which atoms belong to which functional groups. If requested, SparcleQC will automatically create the functional group partitions for each residue and peptide bond to be used in an F-SAPT postprocessing analysis.

SparcleQC also supports relative interaction energy studies for congeneric ligand pairs, which are common in drug design.^37^ When studying congeneric protein:ligand pairs with QM/MM, a fair comparison includes identical QM regions. Realistically, the protein structure associated with each ligand may differ slightly due to either equilibration after the ligand is changed, or the use of different crystal structures. This complicates the creation of two identical QM regions because coordinates (and relative protein:ligand distances) are now different between the two proteins. Our software incorporates a templating algorithm which uses an existing, user-generated QM region from a previous SparcleQC run as a guide to create the QM region of a second, similar but not identical, system. To accomplish this, SparcleQC determines the composition of the previously generated (template) QM region by storing the residue identifiers, and the atomic composition from each residue, that are included in the QM region. Next, each residue in the template protein is mapped to a residue in the new PDB by comparing a neighborhood of five residues. If a match is found, the same atoms of the residue from the template are included in the QM region for the current PDB. If a match cannot be found in the template protein, the residue will be assigned to the QM or MM region solely based on the user-specified cutoff distance. Nonprotein atoms, including solvent or ions, are also handled using the cutoff radius. In our experience, this approach ensures that the QM region remains as similar as possible, while accommodating small differences in coordinates and protonation states. For complete certainty, we recommend users of this feature validate that their QM regions are similar via visual inspection. An example of this system preparation for a pair of proteins with congeneric ligands is given in our documentation.

## Example Calculations

SparcleQC is intended for users of electronic structure theory packages who desire QM/MM energies of protein:ligand systems, but are inhibited by the tedious and labor-intensive system setup. In the following, we demonstrate the utility of SparcleQC by computing protein:ligand interaction energies of QM/MM systems generated by SparcleQC, and comparing those interaction energies to a fully-QM reference energy.

### Basic Use

To test the effectiveness of the SparcleQC workflow, we computed a reference interaction energy of thioredoxin, an enzyme that helps to maintain intracellular redox potentials, and the ligand 2-(*n*-morpholino)-ethanesulfonic acid (PDB 1ABA^38^). This system was chosen due to its relatively small size (1497 atoms), allowing for a single-point QM interaction energy on the entire protein. The protein and ligand were prepared with Chimera X version 1.10 where all waters that form at least two hydrogen bonds with the protein or are near the ligand are retained; the structure is provided in the Supporting Information. Using Psi4 version 1.8 and the HF-3c^39^ method with the MINIX basis set, we calculated the fully-QM interaction energy as −24.81 kcal mol^–1^. The three fully-QM jobs [including (1) all of the ligand, (2) all of the protein, and (3) all of the protein and ligand atoms from the PDB at the QM level] accumulated a wall time of 6.5 days on 10 cores of a Xeon 6226 CPU. HF-3c is a low-cost, minimal-basis method designed to enable efficient calculations on large systems. While it is not as accurate as higher-level DFT or ab initio methods, it is sufficient for detecting relative trends in interaction energies. Note that interaction energies, defined above, give the bare strength of attraction or repulsion between the protein and ligand when they are in their complex geometry; they are only one component of the binding energy, which also includes protein–water and ligand-water interactions. For this reason, interaction energies tend to be much larger in magnitude than binding energies. In SparcleQC, any waters included in the PDB file are accounted for as part of the protein for interaction energy purposes.

We then used SparcleQC to create Psi4 QM/MM input files with a 5 Å cutoff, as this returns a QM region of reasonable size for our system and preferred computational intensity (235 protein and water atoms). We applied the balanced redistributed charge scheme (BRC),^6^ which performed well in previous studies.^7^ The systems in the resulting three Psi4 input files generated by SparcleQC are (1) the 25-atom ligand, (2) the protein with 235 QM atoms the rest of the protein as ff19SB^17^ point charges, and (3) the QM/MM complex. For this representation of the complex, the HF-3c/MINIX interaction energy is −23.74 kcal mol^–1^, only 1.1 kcal mol^–1^ higher than the fully-QM reference energy (−24.81 kcal mol^–1^). This accuracy was achieved in just 12 min, compared to 6.5 days for the fully-QM calculation.

### Automation with Python API

In our basic example, we only presented one QM/MM interaction energy. A more thorough study would include testing the convergence of the interaction energy as the QM region increases in size. To prepare input files with increasingly larger QM regions, we can create a Python loop that will generate a SparcleQC input dictionary for multiple requested cutoffs (increasing distances from the seed, to grow the QM region). Within the Python loop, we also run SparcleQC, as shown in Figure 3.

**Figure 3:**
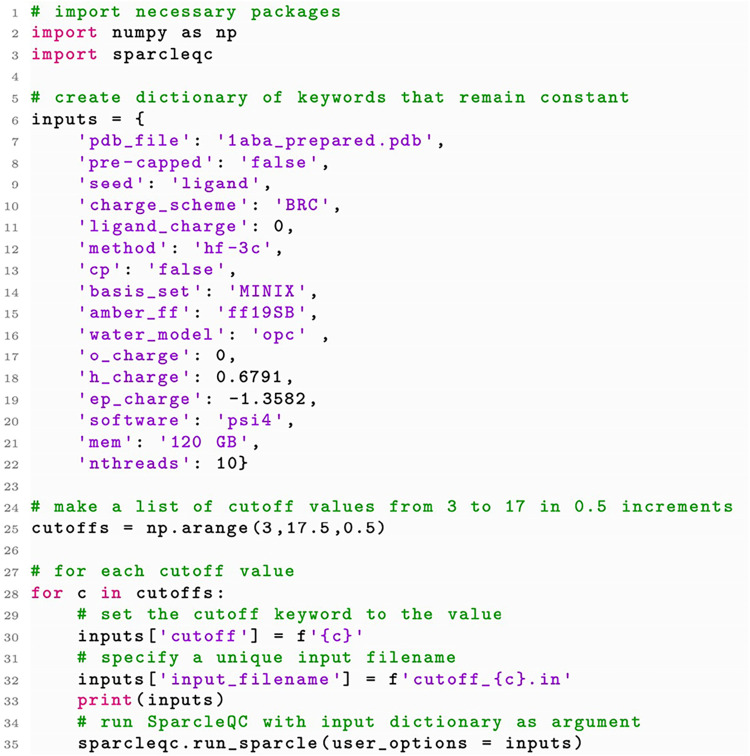
Python script used to generate QM/MM input files with increasingly larger QM regions. A dictionary of options, ‘inputs’, is defined with keywords that remain constant throughout the procedure. Cutoffs are declared as a list of values that range from 3 to 17 (Å), in increments of 0.5. For each cutoff, a new dictionary is created with the cutoff specified and a unique input filename. Finally, SparcleQC is run for each cutoff.

Running this script generates directories for each of the cutoffs, containing protein, ligand, and complex Psi4 input files, among other files generated by SparcleQC. In this example, the QM system size ranges from 167 to 1259 protein atoms. We use the Psi4 files to compute interaction energies for systems of increasing QM-cluster size and the rest of the protein represented by point charges (specifically electrostatic embedding), and the results are shown in Figure 4. With a 5.5 Å cutoff, the QM/MM interaction energy was −24.51 kcal mol^–1^, within 0.5 kcal mol^–1^ of the fully-QM reference (−24.81 kcal mol^–1^). This QM/MM calculation involved (1) a 25-atom ligand, (2) a 312-atom QM protein embedded in point charges, and (3) a 337-atom protein:ligand complex embedded in point charges. On the same computational resources as the fully-QM system, these calculations accumulated only 20 min of wall time. In contrast, the QM-cluster-only interaction energy (at this 5.5 Å cutoff) without point charges is −19.71 kcal mol^–1^. The the QM-cluster-only interaction energies converge extremely slowly.

**Figure 4:**
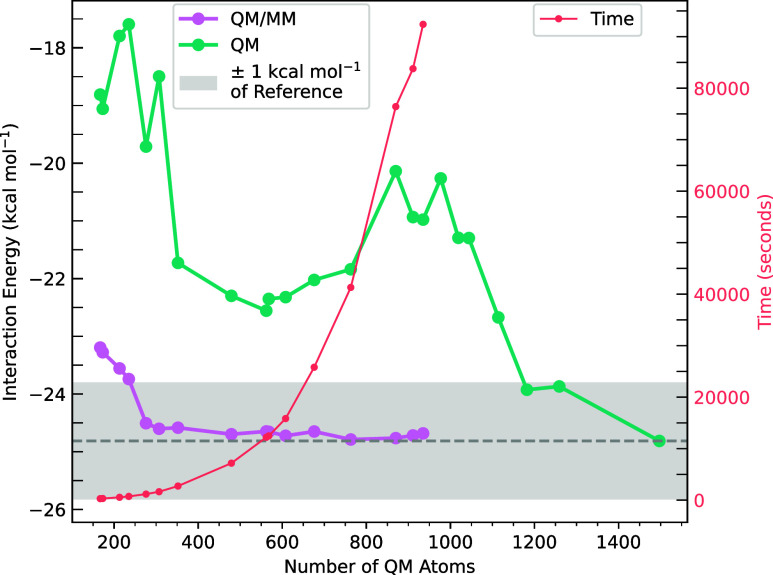
Interaction energy (HF-3c/MINIX) plotted as a function of QM system size for the 1ABA protein:ligand complex. QM refers to QM-cluster-model geometries without point charges. QM/MM refers to the QM-cluster-model geometries with point charges that represent the rest of the protein, created by SparcleQC. The gray dashed line is the reference interaction energy of the fully-QM system, and the gray shaded region represents ± 1 kcal mol^–1^ of the reference energy. Cumulative wall time is plotted for each set of the QM/MM calculations, which is the same as the times for the QM-cluster calculations. The maximum time plotted on this graph is 1 day, and the reference energy was computed in 6.5 days. All timings were completed on 10 cores of a Xeon 6226 CPU.

The same Python syntax shown in Figure 3 could be used for other loops, in the case that a user wanted to process multiple PDB files, test different force fields, or enforce a convergence study that stops at a certain QM region size, for example. We have used a similar loop, this time through software programs, to prepare input files for NWChem and Q-Chem that include the same system setup as the QM/MM file in our basic Psi4 example. For all three codes, the SCF convergence was set to 10^–6^, and default settings were used otherwise. For this example, we computed the interaction energies with Hartree–Fock, the STO-3G^40^ basis set, and the counterpoise correction. This level of theory is not really suitable for production use, as it lacks dispersion interactions, but allowed for a quick check of consistency between the different quantum chemistry programs as called through SparcleQC. The codes returned interaction energies of 2.86 (Psi4), 2.84 (NWChem), and 2.84 kcal mol^–1^ (Q-Chem). We consider this very satisfactory, considering that many default settings were used for each of the codes, which may disagree. Users can modify program-specific keywords via SparcleQC options, which are listed in the documentation.

## Conclusions

The setup of QM/MM input files for protein:ligand systems is often tedious, due primarily to the large number of atoms and the covalent bonds that cross the QM/MM region. SparcleQC automates this process by generating QM/MM-like input files from a PDB file and a Python dictionary of options. Our Python package carves out and caps the QM region, obtains point charges, and creates input files for Psi4, NWChem, and Q-Chem, offering nine schemes for altering the QM/MM boundary charges.

SparcleQC’s command-line operation and Python API enable high-throughput file creation from user-defined parameters, enhancing workflow consistency and efficiency. Furthermore, SparcleQC verifies integer charges at multiple stages, ensuring that the final input file generated has integer charges in both the QM and MM regions (if a balanced or distributed charge scheme is used).

The resulting input files may be used for energy calculations of the protein, ligand, and the protein:ligand complexes separately, preparing for interaction energy calculations. We have shown promising results for computing an interaction energy with HF-3c within 0.5 kcal mol^–1^ of the 1497-atom system reference in just 20 min (wall time) rather than 6.5 days on the same computational resources. Furthermore, SparcleQC can create SAPT files for direct interaction energy calculations, like those in ref 7. We have also introduced features such as automatic functional group partitioning for F-SAPT and QM-region matching for creating QM/MM files of similar protein:ligand complexes. These features enable seamless setup for studies of relative protein:ligand interaction energies of congeneric ligands, as well as studies of conformational energy changes during a molecular dynamics trajectory.

Currently, SparcleQC is designed specifically for protein: ligand systems and relies on the presence of a protein backbone to define and partition the QM and MM regions. As such, the framework is not directly applicable to systems that lack a protein backbone, such as host–guest or DNA-ligand interactions. Extending SparcleQC to support these systems would require methodological adaptations, which is an area for future development.

## Supplementary Material





## Data Availability

The data that support the findings of this study are available in the article and its Supporting Information. SparcleQC is publicly available on GitHub: https://github.com/carolinesargent/sparcleqc. Documentation can be found at https://sparcle-qc.readthedocs.io/en/latest/

## Notes

PDBs, SparcleQC input files, Psi4 input and output files, and notes on protein preparation for 1ABA are available in the electronic Supporting Information (ZIP)
An explanation of the different available charges schemes is also included, as well as an additional example of QM/MM versus a fully-QM reference calculation (PDF)
