# Supplementary material for: SparcleQC: Automated Input File Creation for QM/MM Studies of Protein:Ligand Complexes
Source: J Chem Inf Model. 2025 Jun 17;65(13):6433–40. doi: 10.1021/acs.jcim.5c00617 (PMC12264949; doi:10.1021/acs.jcim.5c00617)
Supplement: Supplementary file 1 [file ci5c00617_si_001.pdf]

Supporting Information for SparcleQC: Automated Input File Creation for QM/MM Studies of Protein:Ligand Complexes

Caroline S. Glick,† Isabel P. Berry,† and C. David Sherrill*,†,‡

†*School of Chemistry and Biochemistry, Georgia Institute of Technology, Atlanta, GA*

‡*Center for Computational Molecular Science and Technology, Georgia Institute of Technology, Atlanta, GA*

E-mail: sherrill@gatech.edu

Available Charge Schemes

Table S-1: Charge schemes to change point charges at the boundary of the QM and MM regions. q refers to charges. M1, M2, and M3 refer to atoms 1, 2, and 3 bond lengths away from the cut bond (shown in Figure 1 of the main text). q_{bal} is the charge required to return the MM residue at the boundary to an integer charge.

Charge Scheme	Charge Manipulation	Charge Distribution
Z1	$q_{\mathrm{M1}} = 0$	none
Z2	$q_{\mathrm{M1}} = q_{\mathrm{M2}} = 0$	none
Z3	$q_{\mathrm{M1}} = q_{\mathrm{M2}} = q_{\mathrm{M3}} = 0$	none
DZ1	$q_{\mathrm{M1}} = 0$	add q_{bal}/N to the N atoms in the residue
DZ2	$q_{\mathrm{M1}} = q_{\mathrm{M2}} = 0$	add q_{bal}/N to the N atoms in the residue
DZ3	$q_{\mathrm{M1}} = q_{\mathrm{M2}} = q_{\mathrm{M3}} = 0$	add q_{bal}/N to the N atoms in the residue
BRC	$q_{\mathrm{M1}} = 0$	add q_{bal}/N to midpoints of the N M1-M2 bonds within same residue
BRC2	$q_{\mathrm{M1}} = 0$	add q_{bal}/N to the N M2 atoms within same residue
BRCD	$q_{\mathrm{M1}} = 0$	add $2 * q_{bal}/N$ to the midpoints of the N M1-M2 bonds then subtract (q_{bal}/N) from each M2 within the same residue

Another Example: 1AAL

We present a QM/MM vs QM-cluster study of a disulfide mutant of basic pancreatic trypsin inhibitor (PDB 1AAL) with a phosphate ligand. We have neutralized the ligand to aid in SCF convergence. Hydrogens were added to the protein in ChimeraX 1.10, and all waters were removed except those that had two or more hydrogen bonds to the protein or are near the ligand. The procedure follows the 1ABA example in the main text. The PDB and SparcleQC input files are in the electronic Supplementary Material.

Table S-2: 1AAL interaction energies (kcal mol^{-1}) computed with HF-3c in Psi4. Atoms refer to the number of atoms in the QM region of the protein. For QM/MM, the rest of the protein is represented by ff19sb point charges. No external point charges were used to compute energies in the QM column. Cutoff refers to the cutoff used in the SparcleQC input file. The reference energy (fully QM, including all protein and ligand atoms) is -29.66 kcal mol^{-1}.

Atoms	Cutoff	QM/MM	QM
53	3.0	-29.565	-32.818
65	3.5	-29.938	-33.583
106	4.0	-29.977	-34.753
184	4.5	-29.775	-33.698
225	5.5	-29.869	-32.859
253	7.0	-29.916	-34.316
292	7.5	-29.768	-33.522
295	8.0	-29.728	-33.136
420	8.5	-29.398	-32.369
